# Gender-Based Disparities in the Evaluation and Management of Acute Coronary Syndromes

**DOI:** 10.7759/cureus.107483

**Published:** 2026-04-21

**Authors:** Jami Rance, Marc M Kesselman

**Affiliations:** 1 Medicine, Dr. Kiran C. Patel College of Osteopathic Medicine, Nova Southeastern University, Tampa, USA; 2 Rheumatology, Dr. Kiran C. Patel College of Osteopathic Medicine, Nova Southeastern University, Davie, USA

**Keywords:** acute coronary syndromes (acs), atypical cardiac symptoms, cardiovascular disease in women, door-to-electrocardiogram time, implicit bias, sex disparities

## Abstract

Cardiovascular disease remains one of the leading causes of mortality globally. Disparities in identifying the presence of cardiovascular disease during clinical evaluation between men and women are likely to contribute to high morbidity and mortality rates, especially in the emergency department setting. Specifically, compared with their male counterparts, women tend to present with atypical and non-specific symptoms, which may lower clinical suspicion and lead to significant delays in diagnosis, especially in obtaining an electrocardiogram (ECG). In addition, women often demonstrate lower help-seeking behaviors when symptoms occur, slowing their initial presentation to the emergency department, which may be influenced by a fear of dismissal, even when their presenting symptoms are typical of those reported by men. Multiple delays in diagnosis can have compounding effects on treatment, which may lead to a longer time to revascularization when needed after the onset of symptoms. These multifactorial delays have been shown to be associated with adverse outcomes unique to women, including higher complication rates, longer hospital stays, and poorer short-term outcomes. Contributing factors associated with these delays may involve a confluence of factors, including but not limited to the historical lack of cardiovascular research focused on women, limited symptom recognition among both patients and clinicians, and the potential influence of bias in clinical decision-making. Addressing these factors should begin with standardized emergency department triage protocols that incorporate symptom recognition specific to men and women, which will aid in prompt ECG acquisition when necessary. Standardized training to increase awareness of the differences in presentation of cardiovascular disease among men and women is warranted. In addition, further research efforts are necessary to achieve meaningful improvements in timely, evidence-based cardiovascular care for women. These disparities are likely multifactorial, reflecting the relationship between the differences in symptom presentation, comorbidity burden, healthcare access, and system-level processes, rather than any single causal factor.

## Editorial

Cardiovascular disease is the leading cause of mortality worldwide, particularly among women. Accumulating evidence suggests that women experience profound inequities in emergency department evaluation and management, a critical point in timely diagnosis. Differences between men and women in the management of acute chest pain have been consistently documented, with women often receiving less aggressive early intervention despite at times presenting with similar complaints as men [1,2]. Meanwhile, women with acute coronary syndromes most commonly present with non-classic and non-specific symptoms such as dyspnea, vomiting, and jaw discomfort, which deviate from the traditional symptomatology more frequently observed in men [1,3]. Studies suggest that young women are at high risk for underdiagnosis due to the combination of non-classic presentation and the availability bias that cardiac arrest occurs primarily in older adults [3]. As a result, women presenting without chest pain are more likely to have their symptoms attributed to non-cardiac causes, further contributing to delays in diagnosis and management [1,4].

The standard of care for all patients presenting with symptoms associated with cardiac ischemia is a door-to-electrocardiogram (ECG) time of less than 10 min, a critical benchmark for the timely diagnosis of acute myocardial infarction [4]. However, evidence suggests that women experience significant delays in meeting this clinical standard. In a cohort of patients with a confirmed diagnosis of acute myocardial infarction, men received an electrocardiogram within a mean of 6.7 min compared with 12.7 min in women, a delay that exceeds recommended time targets [4]. This pattern highlights a critical vulnerability for women, whose ischemic symptoms may be less readily recognized as cardiac in origin [1]. Because early ECG evaluation determines eligibility for time-sensitive therapies, even modest delays at presentation may have downstream effects on treatment decisions and outcomes [2].

Differences in symptom presentation may partially explain these delays. Compared with their male counterparts, women are reportedly less likely to recognize and report ischemic symptoms or seek medical attention promptly [3]. Studies further suggest that women tend to minimize their symptoms due to fear of dismissal by medical staff or attempts to self-medicate, especially when symptoms are non-specific [3]. Symptoms, including anxiety, depression, and psychological distress during a cardiovascular event, may mask underlying ischemic symptoms and lead to misattribution to stress or anxiety [5]. Overall, these prehospital factors further prolong the diagnosis of acute coronary syndromes and compound downstream disparities for women [1].

In addition, the internal implicit bias of clinicians may be one of several factors contributing to late diagnosis. In a small qualitative study of 14 women with myocardial infarction, participants described perceived gender- and age-based bias that they felt contributed to delays in care [3]. Specifically, differences in triaging men and women need to be prioritized, which may be partially influenced by unconscious biases shaped by existing knowledge of cardiovascular risk and symptom presentation [2]. It is important to emphasize that bias is unlikely to be a primary influence on these disparities and instead likely interacts with other factors, including differences in symptom presentation, comorbidity burden, and system-level processes [1,2].

Delays and deviations from the standard of care are not without consequences, as women experiencing acute myocardial infarction are more likely to have complications, worse early outcomes, and longer hospital lengths of stay [2]. It is also noted that women with confirmed myocardial infarction are less likely than men to receive revascularization via coronary angiography within 30 days of diagnosis [1]. In addition, women are more likely to experience longer hospital stays and higher rates of complications compared to men, especially among women younger than 55 years old [2]. From 2000-2009, women experienced a 30-day mortality rate of 16.4%, which is significantly higher than the recorded 11.7% rate of men [2]. These higher rates of negative outcomes for women are associated with increased healthcare costs, complications such as infections, and further patient distress [2]. In addition, women who experience anxiety and depression following hospitalization for myocardial infarction are also more likely to experience increased rehospitalization, reduced quality of life, and worse overall recovery [5]. Emerging studies also suggest that non-cardiac-related health factors, such as anxiety and depression, could be contributing to poorer outcomes for women post-acute myocardial ischemia (AMI) [2].

Compared to their male counterparts, young women (ages 21-55 years old) were more likely to present to the emergency department with more comorbidities such as diabetes and hypertension [2]. In this population of women, they experienced a 30-day mortality of nearly 45% higher than that of males of the same age during 2000-2009 [2]. It could be suggested that a young age in conjunction with non-classic presentation would lead to significant underdiagnosis; however, over 80% of young women did report chest pain as an inciting symptom to their ED visit [2]. This finding challenges the previous idea that non-classic symptom presentation alone accounts for diagnostic delays in women, suggesting that systemic factors may play a more significant role than previously acknowledged. Bridging this gap in care begins with further pursuit of women’s cardiovascular health research and the implementation of standardized emergency care protocols for both men and women. Women, particularly young women, have historically been underrepresented in cardiovascular clinical trials, further limiting the development of diagnostic criteria and risk stratification tools that account for non-chest pain symptom patterns in women [2].

While the causes of disparities in the emergent care of women in the setting of cardiac complaints are multifactorial, they are not fixed in nature. In addition to further research, emergency departments could consider implementing triage protocols that screen for gender-specific cardiac symptoms, such as a structured decision-making pathway that incorporates non-specific symptom presentations (e.g., nausea, anxiety) to trigger faster ECG acquisition [4]. Implementing protocol-driven care can reduce variability in clinical judgment and allow for more timely diagnosis and treatment for women comparable to that provided to men [1]. Specifically, healthcare workers in the emergency department could benefit from structured implicit bias training, such as case-based simulation, audit-and-feedback on triage decisions, and standardized clinical decision support tools [2]. In the setting of a time-sensitive disease, it is essential that institutional changes are implemented to ensure that women receive equitable cardiovascular care at the onset of symptom presentation. These interventions can be incorporated within the existing emergency department workflow, including triage checklists and electronic health record-based prompts for ECG acquisition.

Overall, disparities in the evaluation and management of acute coronary syndromes among women remain significant and are associated with worse outcomes [1,3]. Although these disparities have been well documented, their persistence highlights a gap between current research and clinical practice that warrants targeted, system-level, and clinician-level interventions. Moving forward, establishing standardized clinical guidelines that account for non-classic symptoms, furthering women’s cardiovascular health research, and initiating bias training for healthcare professionals could help improve outcomes for women, similar to those seen in the diagnosis and treatment of men [1,2]. Without significant change in the diagnosis and treatment of women presenting with non-specific symptomatology associated with cardiovascular disease in the emergency department, morbidity and mortality among this patient population will continue to rise, outcomes that could otherwise be prevented [2]. Clinically, this highlights the importance of maintaining a high level of suspicion for acute coronary syndromes in women presenting with non-chest pain symptoms to ensure implementation of timely ECG regardless of symptom presentation.
